# Pathways to psychiatric care in South India and their socio-demographic and attitudinal correlates

**DOI:** 10.1186/1753-6561-6-S4-P13

**Published:** 2012-07-09

**Authors:** S Faizan, BN Raveesh, LS Ravindra, K Sharath

**Affiliations:** 1 Department of Psychiatry, Mysore Medical College and Research Institute (MMC&RI), India

## Introduction

An understanding of the pathways to psychiatric care is critically important as a means of informing national policy and re-directing the meager resources towards better management of psychiatric patients. Since pathways to psychiatric care in South India are poorly understood we conducted a study in Mysore, South India to determine the various pathways to Psychiatric care. The aim is to determine the health-seeking behaviour of mentally ill patients and the attitude to mental illness and its treatment in Mysore, South India.

## Methods

A cross sectional hospital based study was conducted among 104 consecutive, newly registered consenting patients/informants presenting to the Outpatient clinic of a Tertiary hospital in Mysore over 2 months. A Pre tested, Semi structured Interview based on a translated version of the WHO Encounter form was used to obtain Socio-Demographic details and referral patterns and a Pre Validated Modified Opinion of Mental Illness Scale was used to obtain information regarding their attitude to mental illness. Diagnosis was made based on ICD-10. The Chi square test, t test and Kruskal Wallis test were used to analyze the data.

## Results

Allopathic practitioners (AP's) were the first carers for 51.0 %; Religious healers (RH's) for 26.9 %, while only 22.1 % consulted Mental Health Professional (MHP) as First carer. An average delay of 26.12 weeks was seen from the onset of illness to presentation at our clinic. The longest delay was associated with patients who had AP's as first carer [38.94 weeks (mean)] while the patients directly consulting an MHP had the least delay [4.69 weeks (mean)]. The first carers were consulted largely at the suggestion of family members and relatives (66.3%). The appearance of RH's in the pathway was associated with greater superstitious beliefs about mental illness (*P*<0.001), rural residence (*P*<0.05) and lower educational attainment (*P*<0.05). Treatment costs (49 %) and confidentiality concerns (35.6%) were reported as major barriers to obtaining psychiatric care.

## Conclusions

Three Major Pathways including referral by AP's, pathway through RH's and direct consultation of MHP were seen. The study highlights areas of importance requiring attention in order to create a broad community based mental health care system. These areas include the education of AP's and RH's regarding mental illness, education programs to counter the stigma, superstitions and misconceptions surrounding mental illness, counseling family members of psychiatric patients and addressing concerns regarding confidentiality and treatment costs.

## Limitations

Since the information was gathered retrospectively recall bias may influence results to some extent.

